# CDX-301 prevents radiation-induced dysregulation of miRNA expression and biogenesis

**DOI:** 10.1016/j.omtn.2022.11.010

**Published:** 2022-11-15

**Authors:** Dharmendra Kumar Soni, Vidya P. Kumar, Shukla Biswas, Gregory P. Holmes-Hampton, Sharmistha Bhattacharyya, Lawrence J. Thomas, Roopa Biswas, Sanchita P. Ghosh

**Affiliations:** 1 Department of Anatomy, Physiology and Genetics, School of Medicine, Uniformed Services University of the Health Sciences, Bethesda, MD 21045, USA; 2 Armed Forces Radiobiology Research Institute, Uniformed Services University of the Health Sciences, Bethesda, MD 20889, USA; 3 Celldex Therapeutics, Inc., Fall River, MA 02723, USA

**Keywords:** MT: Non-coding RNAs, acute radiation syndrome, CDX-301, irradiation, microRNA, radiation countermeasures

## Abstract

Risks of radiation exposure necessitate the development of radioprophylactic drugs. We have reported the efficacy of CDX-301, a recombinantly developed human protein form of Fms-related tyrosine kinase 3 ligand (Flt3L), as a radioprophylactic and radiomitigatory agent. Here, we performed global microRNA profiling to further understand the mechanism of action of CDX-301. We find that CDX-301 administration 24 h prior to total body irradiation prevents radiation-induced dysregulation of microRNA biogenesis and expression in murine serum and spleen samples in a time- and tissue-dependent manner. Further analysis shows that activation of the HOTAIR regulatory pathway has a prominent function in radiation-induced injury responses, which is inhibited by pre-treatment with CDX-301. Moreover, CDX-301 attenuates radiation-induced dysregulation of several cellular functions such as inflammatory and immune responses. In corroboration, we also find that pre-treatment with CDX-301 restores the expression of bone marrow aplasia markers and inflammatory cytokines and growth factors, as well as the expression of genes associated with MAP kinase and TGF-β pathways that are altered by radiation. Our findings provide new insights into CDX-301-mediated molecular and cellular mechanisms and point to a possible novel radioprotective drug for the prevention of irradiation-induced injury and hematopoietic acute radiation syndrome.

## Graphical abstract

**Figure:**
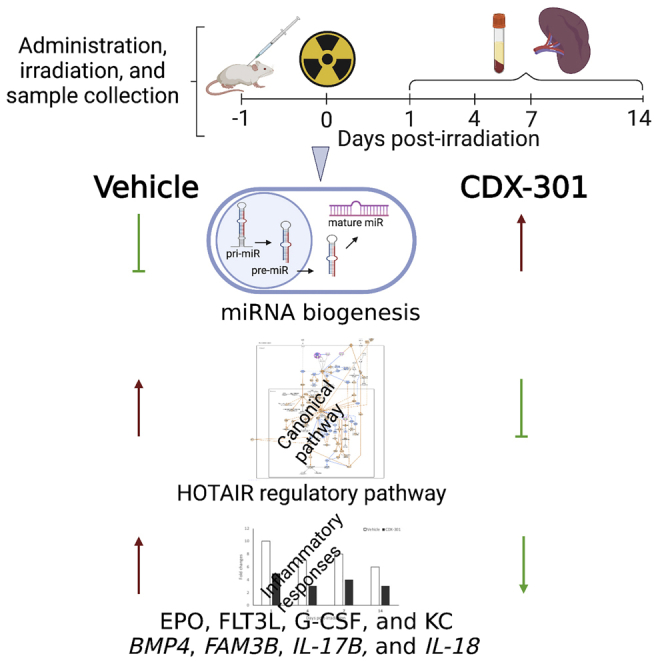

## Teaser

This study identifies that CDX-301 averts radiation-induced lethal responses through prevention of dysregulated microRNA biogenesis, inhibition of HOTAIR regulatory pathway and Flt3L, and attenuation of dysregulated cellular functions, thus providing new insights into CDX-301-mediated molecular and cellular mechanisms and pointing to a possible novel radioprotective drug for hematopoietic acute radiation syndrome.

## Introduction

Risks of exposure to radiation, either through unintentional incidents such as radiotherapy and industrial calamity or intentional terrorist assaults and military actions, are irrefutable.^1^^,^^2^^,^^3^^,^^4^ High-level ionizing radiation exposure even within a short time frame poses serious health hazards leading to tissue/cutaneous injury and, eventually, multi-organ (bone marrow/hematopoietic, gastrointestinal, cardiovascular, and central nervous system) failure that is commonly called acute radiation syndrome (ARS).^5^ Presently there are four US Food and Drug Administration (FDA)-approved drugs available for the treatment of ARS patients: filgrastim (Neupogen), pegfilgrastim (Neulasta), sargramostim (Leukine), and romiplostim (Nplate).^6^ However, these treatments have limitations in that they can only be used 24 h after acute radiation exposure for the mitigation of ARS symptoms. In the aftermath of an accident or an attack, warfighters and first responders are likely to enter the contaminated areas for search and rescue, clean-up, and other operational efforts. Hence, it is essential to develop prophylactic countermeasures that have broad-spectrum therapeutic management. Ideally, these countermeasures would have a wide operational window of administration and could be used in both scenarios, i.e., before and after exposure.

Over the last decades, researchers have studied a number of compounds, such as BBT-059 (a pegylated construct of the 20 kDa protein IL-11),^7^^,^^8^^,^^9^ CBLB613 (a naturally occurring mycoplasma-derived lipopeptide ligand for Toll-like receptor 2/6),^10^ Ex-Rad (4-carboxystyryl-4-chlorobenzylsulfone),^11^ and GT3 (a vitamin E analog of γ-tocotrienol),^12^^,^^13^^,^^14^^,^^15^ as potent radioprotectors for ARS, and these are in different stages of development. Among the four FDA-approved medical countermeasures for ARS, all received approval for other indications: pegfilgrastim and filgrastim for neutropenia, sargramostim for acute myeloid leukemia, and romiplostim for thrombocytopenia.^16^^,^^17^^,^^18^^,^^19^

CDX-301 (developed by Celldex Therapeutics) is a soluble recombinant human protein form of the Fms-related tyrosine kinase 3 ligand (Flt3L) and has passed the phase 1 clinical trial for hematopoietic stem cell transplantation^20^ and cancer immunotherapy.^21^ CDX-301 is a potential candidate for the prevention and/or alleviation of ARS and its fatal consequences. Numerous studies demonstrated the effectiveness of CDX-301/Flt3L in reconstituting myelopoiesis and lymphopoiesis and indicate its potential to ameliorate long-lasting lymphoid cell deficiencies.^22^^,^^23^^,^^24^^,^^25^^,^^26^^,^^27^^,^^28^^,^^29^ Recently, we evaluated the efficacy of CDX-301 in CD2F1 mice with the administration of a single dose in the time range of 24 h prior to until 24 h post total body irradiation (TBI).^30^ We reported both protection and recovery of myeloid and lymphoid cells in circulation and within the tissue, which resulted in increased survival. These findings establish CDX-301 as a promising radiation countermeasure for both prophylactic and mitigatory administration. Additional investigation is needed to understand its mechanism of action and the cellular and molecular responses through which CDX-301 averts injury and increases survival.

In the modulation of almost every cellular and molecular process, microRNAs (miRNAs, miRs), a family of small (∼19–21 nucleotides) single-stranded non-coding RNAs that post-transcriptionally regulate gene expression either through the target messenger RNA (mRNA) degradation or inhibition of translation, have emerged as key regulators of cellular processes.^31^^,^^32^^,^^33^ Recently the role of miRNAs has been implicated in evaluating the efficacy of drugs, opening a new avenue of “miRNA pharmacogenomics” to study the effects of drug treatment on radiation-induced altered miRNA expression and biological pathways.^34^^,^^35^^,^^36^^,^^37^

In the current study, in an effort to understand the radioprophylactic mechanism of CDX-301, we examined global changes in the expression of miRNAs and associated pathways in mice treated with or without CDX-301 24 h prior to TBI. Our analysis reveals radiation-induced alteration in the expression of miRNAs and processing enzymes (such as AGO2, DGCR8, and DICER1). Notably, CDX-301 administration prior to TBI prevents the impact of radiation exposure on miRNA biogenesis in both serum and spleen samples of mice in a time- and tissue-dependent manner. *In silico* analyses of the canonical pathways indicate that activation of the long non-coding RNA, HOTAIR, regulatory pathway has a prominent function in radiation-induced injury responses in both serum and spleen samples, which is inhibited by pre-treatment with CDX-301. Further analysis stipulates that the pre-treatment with CDX-301 attenuates radiation-induced dysregulation of several cellular functions including inflammatory and immune responses. Consistently, we find that radiation-induced altered protein levels of erythropoietin (EPO), Flt3L, and inflammatory cytokines and growth factors, and expression of genes associated with mitogen-activated protein (MAP) kinase and transforming growth factor β (TGF-β) pathways are restored by pre-treatment with CDX-301. Overall, we demonstrate that CDX-301 administration 24 h prior to TBI impedes radiation-induced dysregulation of miRNA expression and provides new insights into CDX-301-mediated molecular and cellular mechanisms. Collectively, these data along with earlier studies suggest that CDX-301 has a pronounced effect in averting DNA damage and cell-cycle arrest, diminution of cellular radiosensitivity, and protection from hematopoietic damage.

## Results

### Radiation alters expression of miRNAs in a time- and tissue-dependent manner

To explore whether the expression of miRNAs is altered in response to radiation, we performed a comprehensive analysis of miRNA expression profiles in the serum and spleen of non-irradiated and irradiated mice at different time intervals. miRNA expression was analyzed between the mice treated with vehicle 24 h prior to TBI (RV) and controls (naive and non-irradiated mice treated with vehicle [NRV]). All data presented were compared with the NRV control group, since no difference in expression of miRNAs was found between naive and NRV groups.

**Figure 1:**
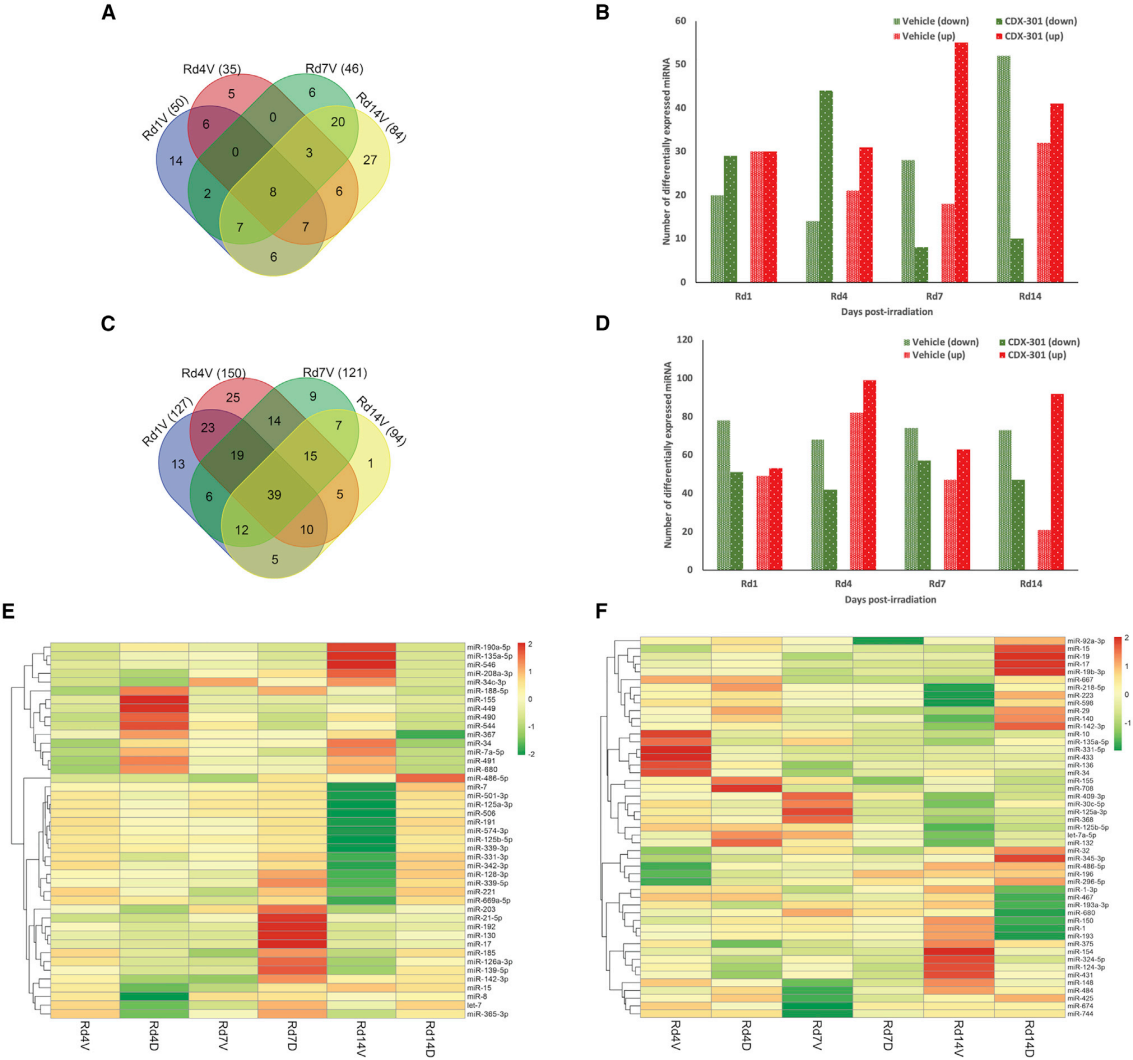
Effect of radiation and impact of pre-treatment with CDX-301 on expression of miRNAs (A) Venn diagram shows a total number of differentially expressed miRNAs in the serum of RV group. (B) Histogram shows a total number of downregulated and upregulated miRNAs in the serum of RV and RD groups compared with the NRV control groups at different time intervals, i.e., on day 1, 4, 7, or 14 post irradiation. (C) Venn diagram shows total number of differentially expressed miRNAs in the spleen of RV group. (D) Histogram shows total number of downregulated and upregulated miRNAs in the spleen of RV and RD groups in comparison with NRV control groups at different time intervals, i.e., on day 1, 4, 7, or 14 post irradiation. (E and F) Heatmaps indicate the differential expression of miRNAs in murine (E) serum and (F) spleen of the RV and RD groups compared with the NRV control group at days 4, 7, and 14 post irradiation.

In mouse serum samples, a total of 50, 35, 46, and 84 miRNAs exhibited altered expression (≥1.5-fold) in Rd1V (day 1 post irradiation in mice treated with vehicle 24 h prior to TBI), Rd4V (day 4), Rd7V (day 7), and Rd14V (day 14), respectively, compared with the NRV group (Figure 1A). On day 1 among 50 miRNAs, 30 were upregulated and 20 were downregulated; on day 4 among 35 miRNAs, 21 were upregulated and 14 were downregulated; on day 7 among 46 miRNAs, 18 were upregulated and 28 were downregulated; and on day 14 among 84 miRNAs, 32 were upregulated and 52 were downregulated (Figure 1B).

Similarly, in murine spleen tissue we identified a total of 127, 150, 121, and 94 miRNAs with altered expressions (≥1.5-fold) in Rd1V, Rd4V, Rd7V, and Rd14V, respectively, compared with the NRV group (Figure 1C). On day 1 among 127 miRNAs, 49 were upregulated and 78 were downregulated; on day 4 among 150 miRNAs, 82 were upregulated and 68 were downregulated; on day 7 among 121 miRNAs, 47 were upregulated and 74 were downregulated; and on day 14 among 94 miRNAs, 21 were upregulated and 73 were downregulated (Figure 1D).

Furthermore, as depicted in Figures 1E and 1F, we also found that those miRNAs that exhibit maximal differential expression on all four days (day 1, 4, 7, or 14) post irradiation in both serum and spleen tissue samples of the RV group compared with the NRV group exhibit variation in expression levels across the days. Overall, these data indicate a significant alteration in the expression of miRNAs in the irradiated mice in a time- and tissue-dependent manner.

### CDX-301 counterbalances the effect of radiation on the expression of miRNAs in murine serum

To investigate the effect of pre-treatment with CDX-301 on irradiated mice, we first compared the miRNA expressions in serum samples of mice treated with CDX-301 24 h prior to TBI (RD group) with the NRV control group at various time intervals. Subsequently, we compared these significantly altered serum miRNAs of the RD group with those of the RV group at different time intervals.

The analyses revealed that the total number of downregulated miRNAs in Rd7V (28) and Rd14V (52) were lower in the Rd7D (day 7 post irradiation in mice treated with CDX-301 24 h prior to TBI) and Rd14D (day 14) groups, i.e., 8 and 10, respectively (Figure 1B). On the other hand, the total number of upregulated miRNAs in Rd7V (18) and Rd14V (32) were higher in Rd7D and Rd14D groups, i.e., 55 and 41, respectively (Figure 1B). This suggests that pre-treatment with CDX-301 reduces the total number of radiation-induced downregulated miRNAs while increasing the total number of upregulated miRNAs in mouse serum samples.

Furthermore, we found that those miRNAs (such as top miRs [miR-490, miR-7a-5p, miR-491, miR-449, miR-34, and miR-680 at day 4 post irradiation, miR-669a-5p, miR-15, miR-342-3p, let-7, miR-17, miR-221, miR-125a-3p, miR-130, miR-128-3p, and miR-142-3p at day 7 post irradiation, and miR-506, miR-125a-3p, miR-339-3p, miR-669a-5p, miR-125b-5p, miR-7, miR-574-3p, miR-191, miR-339-5p, and let-7 at day 14 post irradiation]) which significantly decreased in response to radiation exhibited increased expression on pre-treatment with CDX-301 on the respective days (Figure 1E). This suggests that pre-treatment with CDX-301 suppresses radiation-induced downregulation of miRNA levels in mouse serum samples.

Concurrently, those miRNAs (such as top miRs [miR-8, miR-669a-5p, let-7, miR-125b-5p, miR-365-3p, miR-139-5p, miR-339-3p, miR-185, miR-126a-3p, and miR-192 at day 4 post irradiation, miR-8, miR-155, miR-34c-3p, miR-449, miR-190a-5p, miR-544, and miR-490 at day 7 post irradiation, and miR-491, miR-367, miR-155, miR-188-5p, miR-208a-3p, miR-7a-5p, miR-135a-5p, miR-449, miR-546, and miR-490 at day 14 post irradiation]) which significantly increased in response to radiation showed decreased expression on pre-treatment with CDX-301 on the respective days (Figure 1E). This suggests that pre-treatment with CDX-301 suppresses radiation-induced upregulation of miRNAs in mouse serum samples. The miRNAs that exhibit maximal differential expression in the serum samples from the RV group and their alteration in the RD group on days 4, 7, and 14 post irradiation are represented in the heatmap depicted in Figure 1E. Collectively, these results suggest that pre-treatment with CDX-301 counterbalances the effect of radiation on the expression of miRNAs in mouse serum in a time-dependent manner.

### CDX-301 counterbalances the effect of radiation on the expression of miRNAs in murine spleen

Similarly, to investigate the effect of pre-treatment with CDX-301 in spleen tissues of irradiated mice, we compared the miRNA expression profiles of the RD group with those of the NRV group at different time intervals. Thereafter, we compared the expression of miRNAs that were significantly altered in the spleen tissue of the RD group with those in the RV group at various time intervals.

In spleen samples, analyses of differentially expressed miRNAs between RV and RD groups showed that on days 1, 4, 7, or 14 post irradiation, the total number of downregulated miRNAs, i.e., 74, 64, 72, and 53, respectively, in RV, was lower in Rd1D, Rd4D, Rd7D, and Rd14D, i.e., 51, 42, 57, and 47, respectively (Figure 1D). However, the total number of upregulated miRNAs, i.e., 48, 84, 46, and 20 at days 1, 4, 7, and 14 post irradiation, respectively, in RV were higher in Rd1D, Rd4D, Rd7D, and Rd14D, i.e., 53, 99, 63, and 42, respectively (Figure 1D). This suggests that pre-treatment with CDX-301 reduces the total number of radiation-induced downregulated miRNAs while increasing the total number of upregulated miRNAs in the mouse spleen tissue, similar to that observed in the serum.

Furthermore, the expression of miRNAs (such as top miRs [miR-486-5p, miR-150, miR-296-5p, miR-196, miR-15, miR-32, miR-92a-3p, miR-17, miR-142-3p, and miR-155 at day 4 post irradiation, miR-486-5p, miR-744, miR-19, miR-425, miR-484, miR-19b-3p, miR-375, and miR-136 at day 7 post irradiation, and miR-598, miR-142-3p, miR-17, miR-125b-5p, miR-92a-3p, miR-15, miR-223, let-7a-5p, miR-29, and miR-140 at day 14 post irradiation]) which decreased significantly in response to radiation showed increased expression by pre-treatment with CDX-301 on the respective days (Figure 1F). This suggests that pre-treatment with CDX-301 suppresses radiation-induced downregulation of miRNAs in the mouse spleen similar to that observed in the serum.

Furthermore, the miRNAs (such as top miRs [miR-324-5p, miR-125a-3p, miR-135a-5p, miR-148, miR-368, miR-124-3p, miR-331-5p, miR-433, miR-34, and miR-10 at day 4 post irradiation, miR-1, miR-680, miR-324-5p, miR-30c-5p, miR-409-3p, miR-132, miR-124-3p, miR-368, and miR-10 at day 7 post irradiation, and miR-193a-3p, miR-674, miR-708, miR-193, miR-1-3p, miR-467, miR-431, miR-124-3p, and miR-154 at day 14 post-irradiation]), whose expression significantly increased in response to radiation exhibited decreased expression on pre-treatment with CDX-301 on the respective days (Figure 1F). This suggests that pre-treatment with CDX-301 suppresses radiation-induced upregulation of miRNA expression in the spleen, similar to that observed in the serum. The miRNAs that exhibited maximal differential expression in the spleen tissue samples of the RV group and their alteration in the RD group on days 4, 7, and 14 post irradiation are represented in the heatmap depicted in Figure 1F. Collectively, these findings demonstrate that pre-treatment with CDX-301 counterbalances the effect of radiation on the expression of miRNAs in a time- and tissue-dependent manner in mouse serum and spleen tissues.

Interestingly, we found that the miRNAs miR-125a-3p, miR-125b-5p, miR-135a-5p, miR-142-3p, miR-15, miR-155, miR-17, miR-34, miR-486-5p, and miR-680 were significantly altered in both serum and spleen samples at days 4, 7, and 14 post irradiation. The altered expression of these miRNAs in both serum and spleen suggests a potential global effect of radiation on the associated signaling pathways.

### CDX-301 inhibits HOTAIR regulatory pathways

**Figure 2:**
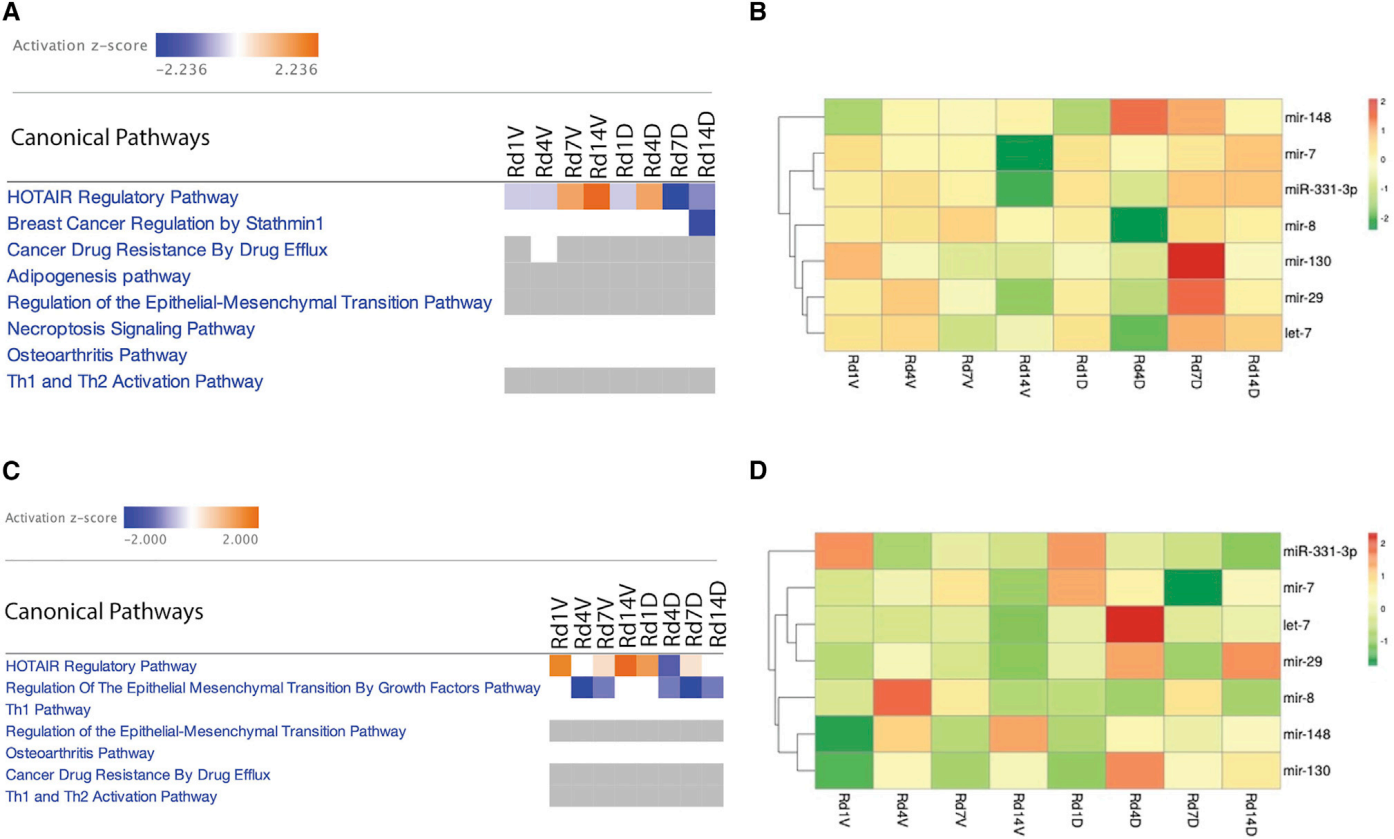
Effect of radiation and impact of pre-treatment with CDX-301 on canonical signaling pathways and related miRNA expressions (A and B) Heatmaps depict (A) the differential regulation of canonical signal transduction pathways and (B) the differential expression of miRNAs involved in the HOTAIR regulatory pathway in the serum of the RV and RD groups compared with NRV control group at different time intervals, i.e., on day 1, 4, 7, or 14 post irradiation. (C and D) Heatmaps show (C) the differential regulation of canonical signal transduction pathways and (D) the differential expression of miRNAs involved in the HOTAIR regulatory pathway in the spleen of the RV and RD groups compared with NRV control group at different time intervals, i.e., on day 1, 4, 7, or 14 post irradiation.

We performed *in silico* analyses of differentially expressed miRNAs at various time intervals in the serum and spleen samples from the RV and RD groups using the Ingenuity Pathway Analysis (IPA) program. As shown in Figures 2A and 2C, the HOTAIR regulatory pathway is the top canonical pathway that is differentially regulated in the serum and spleen from the RV and RD groups in comparison with the NRV group at different time intervals. The results indicate that the HOTAIR regulatory pathway was activated in the serum and/or the spleen samples from the RV group on days 7 and/or 14 post irradiation, which was inhibited by pre-treatment with CDX-301. The heatmap depicts the expression of miRNAs (let-7, miR-130, miR-148, miR-29, miR-331-3p, miR-7, and miR-8) involved in HOTAIR regulatory pathways (Figures 2B and 2D). The analyses of these differentially expressed miRNAs indicate that pre-treatment with CDX-301 restores the expression of these miRNAs.

### CDX-301 modulates upstream signaling molecules in murine serum and spleen

**Figure 3:**
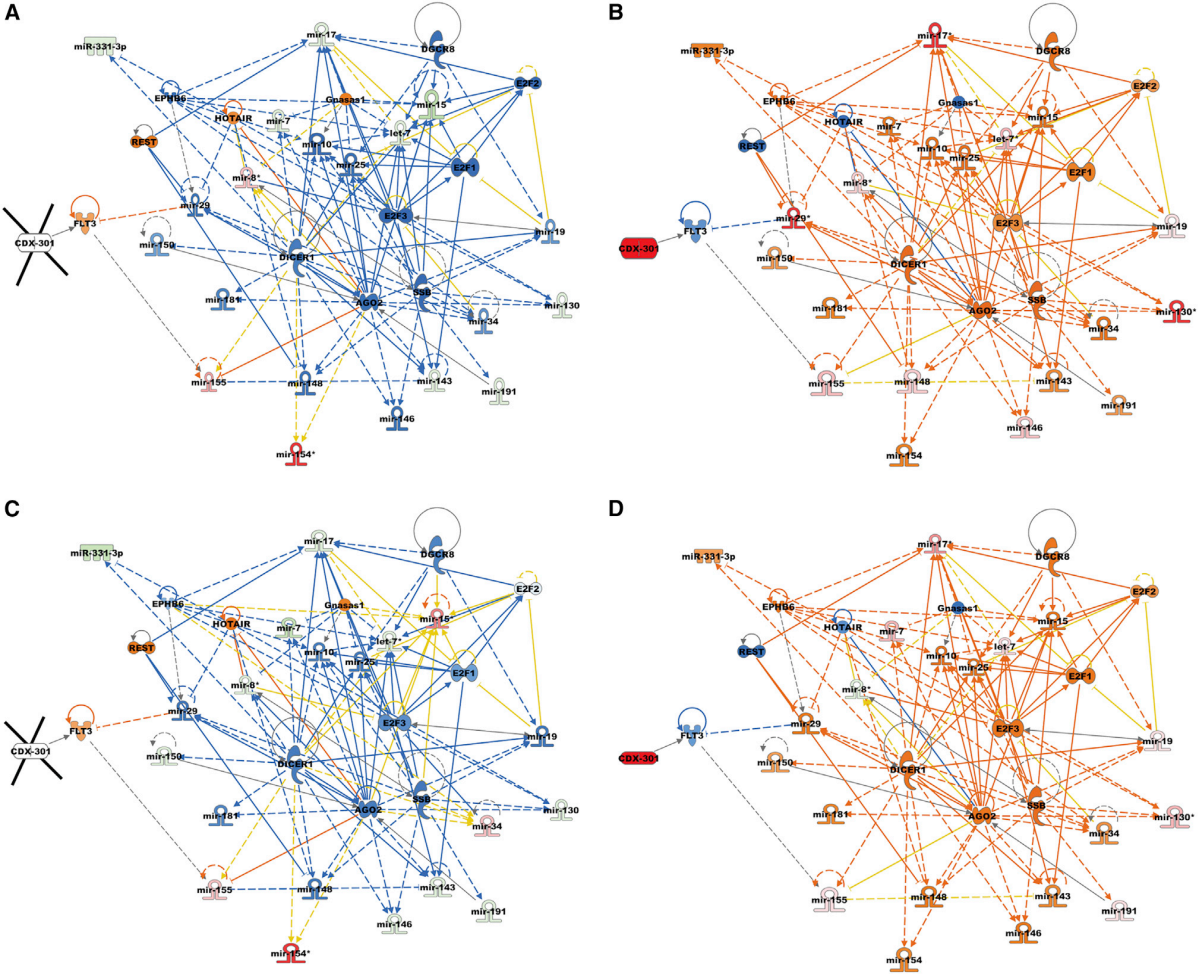
Effect of radiation and impact of pre-treatment with CDX-301 on upstream signaling molecules in the murine serum Upstream regulators are activated and inhibited in (A) Rd7V, (B) Rd7D, (C) Rd14V, and (D) Rd14D groups compared with NRV control groups in the murine serum samples. The color scheme indicates the relative value of the differential expressions and the shapes indicate the type and function, as indicated in Figure S1. Solid arrows represent the genes that interact directly, and dashed arrows represent indirect interactions between genes.

**Figure 4:**
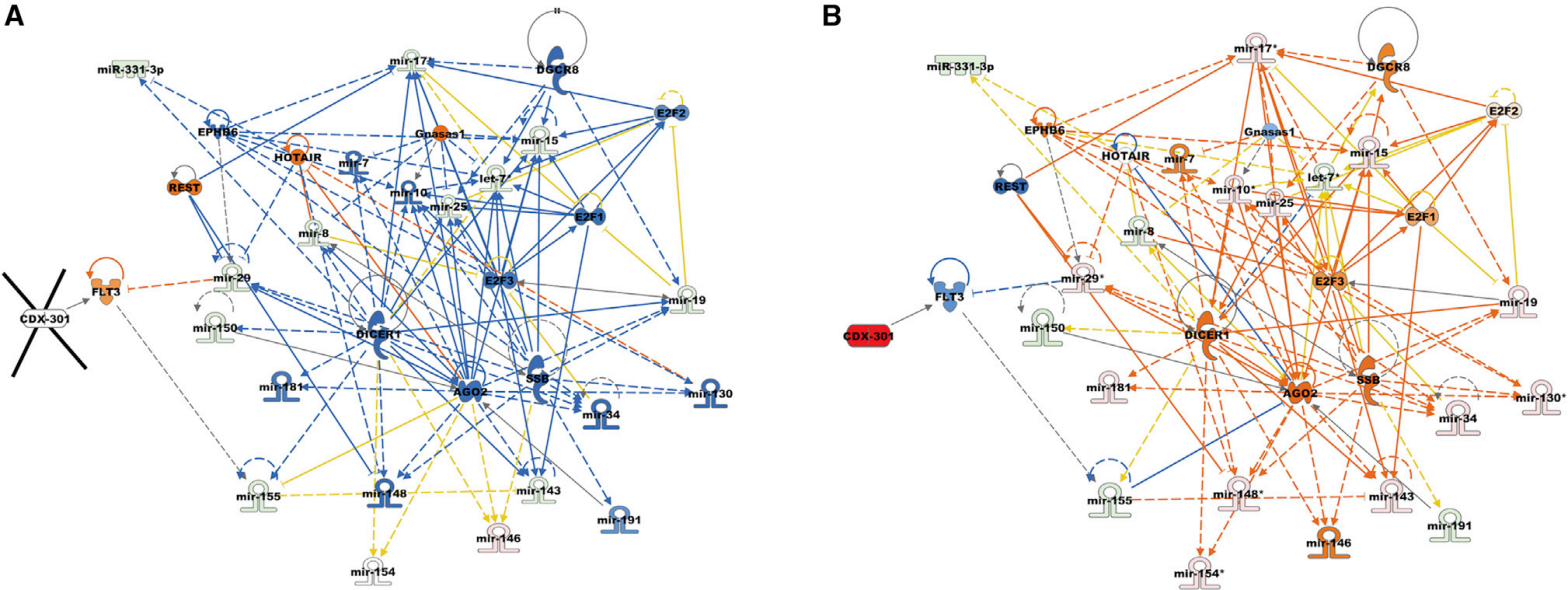
Effect of radiation and impact of pre-treatment with CDX-301 on upstream regulators in the murine spleen Upstream regulators are activated and inhibited in (A) Rd14V and (B) Rd14D groups compared with NRV control groups in the murine spleen samples. The color scheme indicates the value of the differential expressions and the shapes indicate the type and function, as indicated in Figure S1. Solid arrows represent the genes that interact directly, and dashed arrows represent indirect interactions between genes.

To identify regulators that stimulated alterations in gene expression, we performed upstream regulator analyses of differentially expressed miRNAs in the serum and spleen samples from the RV and RD groups. Our analysis showed that in response to radiation, several transcriptional upstream regulators were either activated or inhibited on days 7 and 14 in the serum, and on day 14 in the spleen samples. Interestingly, we find that the functional activities of these transcription regulators were reversed by pre-treatment with CDX-301 (Figures 3 and 4). The *Z* scores of upstream regulators and expression of associated miRNAs in the serum and spleen of the RV and RD groups are listed in Tables S1 and S2. In the RV group, radiation activated the regulatory molecules FLT3, REST, HOTAIR, and Gnasas1, and inhibited AGO2, DGCR8, and DICER1 (molecules that are involved in miRNA biogenesis) in serum and/or spleen samples as depicted in Figures 3A, 3C, and 4A. Additional molecules including E2F1, E2F2, E2F3, and SSB were also inhibited by exposure to radiation in serum and/or spleen samples. However, pre-treatment with CDX-301 caused inhibition of FLT3, REST, HOTAIR, and Gnasas1, and activation of AGO2, DGCR8, DICER1, E2F1, E2F2, E2F3, and SSB in serum and/or spleen samples as depicted in Figures 3B, 3D, and 4B. Collectively, these data demonstrate that treatment with CDX-301 prior to TBI inhibited HOTAIR regulatory pathway and restored miRNA biogenesis.

### CDX-301 modulates cellular function in murine serum and spleen

**Figure 5:**
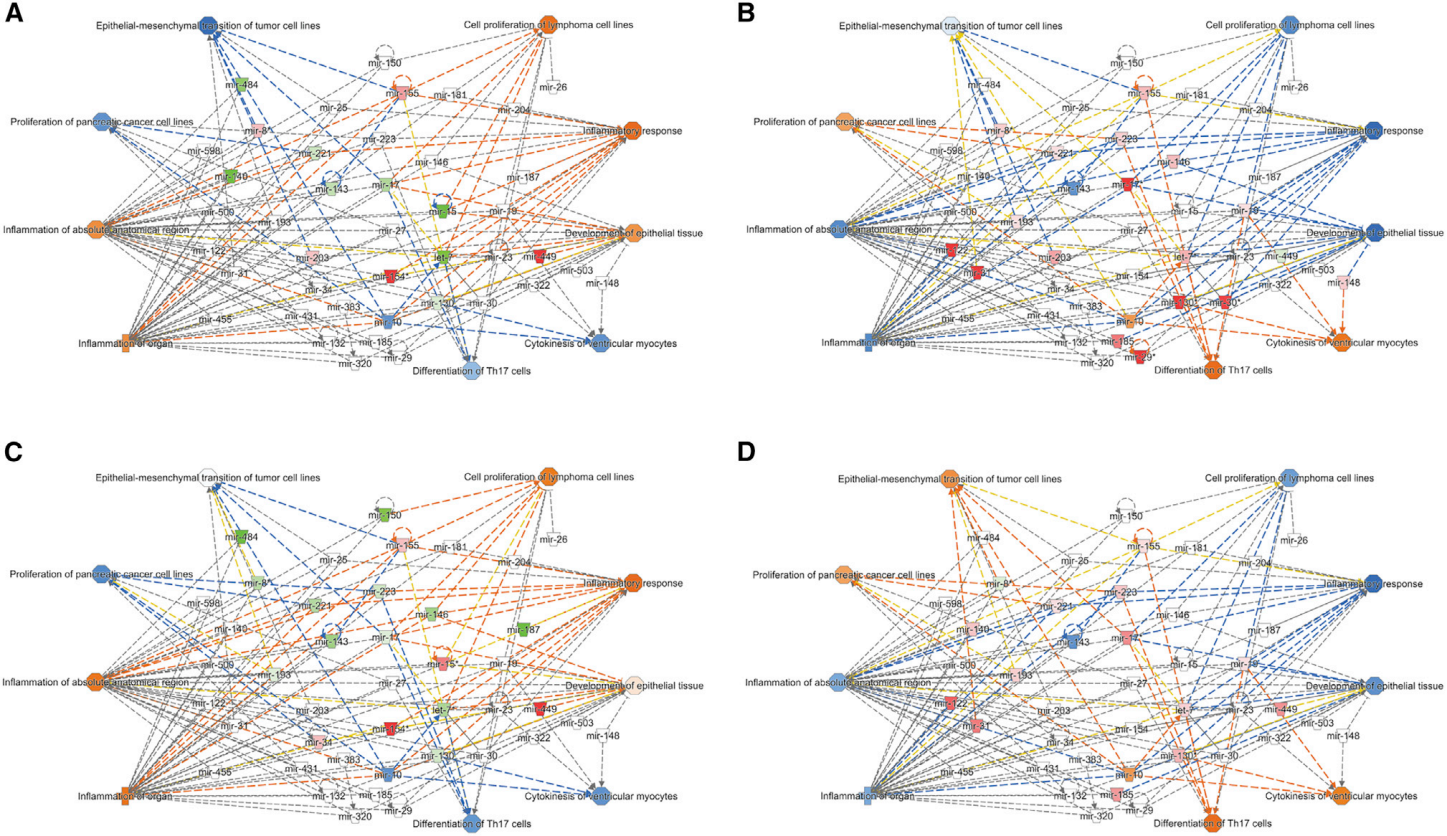
Effect of radiation and impact of pre-treatment with CDX-301 on disease and cellular function in the murine serum Disease and function are activated and inhibited in (A) Rd7V, (B) Rd7D, (C) Rd14V, and (D) Rd14D groups compared with NRV control groups in the murine serum samples. The color scheme indicates the value of the differential expressions and the shapes indicate the type and function, as indicated in Figure S1. Solid arrows represent the genes that interact directly, and dashed arrows represent indirect interactions between genes.

**Figure 6:**
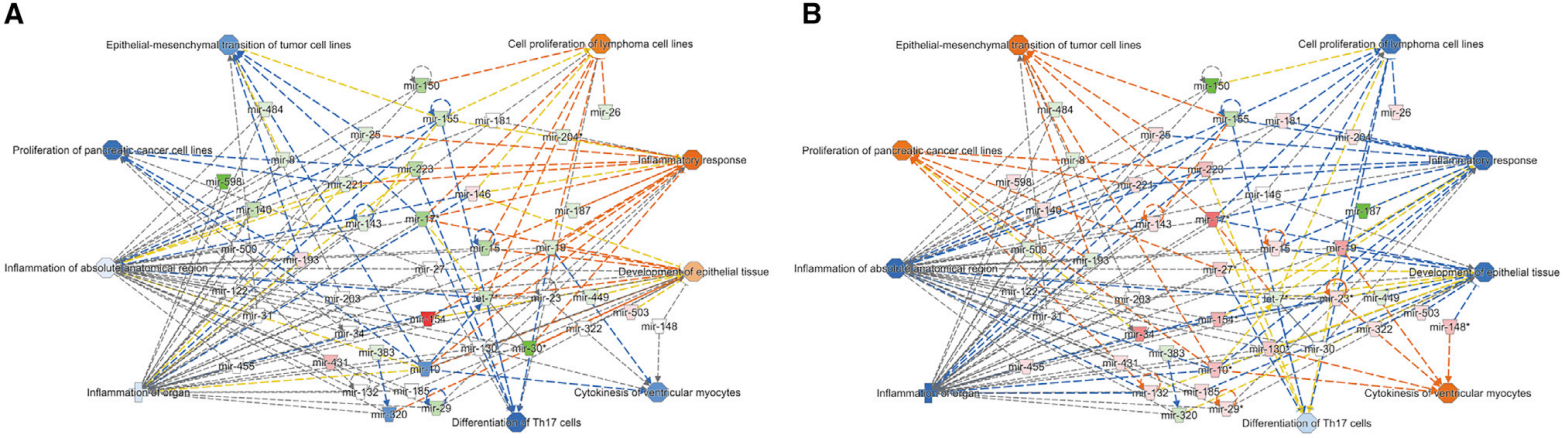
Effect of radiation and impact of pre-treatment with CDX-301 on disease and cellular function in the murine spleen Disease and function are activated and inhibited in (A) Rd14V and (B) Rd14D groups compared with NRV control groups in the murine spleen samples. The color scheme indicates the value of the differential expressions and the shapes indicate the type and function, as indicated in Figure S1. Solid arrows represent the genes that interact directly, and dashed arrows represent indirect interactions between genes.

Subsequently, to identify cellular processes and biological functions that are stimulated by alterations in gene expression, we used IPA to perform disease and cellular function analysis for differentially expressed miRNAs in the serum and spleen samples from the RV and RD groups. The impact of radiation and pre-treatment with CDX-301 on the expression of miRNAs and associated diseases and cellular functions are listed in Table S3. Our analysis showed that in response to radiation, several cellular functions were either activated or inhibited in the serum on days 7 and 14 and in the spleen samples on day 14. Interestingly, our analysis revealed that the functional activities of these cellular functions were reversed by pre-treatment with CDX-301 (Figures 5 and 6). Exposure to radiation activated cell proliferation of lymphoma cell lines, inflammatory responses, and development of epithelial tissue, and inhibited cytokines of ventricular monocytes, proliferation of pancreatic cell lines, and epithelial-mesenchymal transition of tumor cell lines at days 7 and 14 post irradiation in serum and at day 14 post irradiation in spleen (Figures 5A, 5C, and 6A). CDX-301 administration prior to TBI attenuates these various cellular functions, thus protecting animals from radiation-induced lethality (Figures 5B, 5D, and 6B).

Additional functions including inflammation of absolute anatomical region and inflammation of organ pathways that were activated in serum samples from the RV group on both days 7 and 14 post irradiation were attenuated by pre-treatment with CDX-301. Moreover, the differentiation of T helper 17 (Th17) cells, which is inhibited in serum samples from the RV group, was activated by pre-treatment with CDX-301 on days 7 and 14 post irradiation (Figures 5 and 6). Our data suggest that pre-treatment with CDX-301 modulates disease and cellular functions through the regulation of miRNA expression in both serum and spleen tissues.

### CDX-301 attenuates radiation-induced increased levels of EPO and Flt3L in murine serum

To further assess the effect of pre-treatment with CDX-301 on the radiation-induced damage, we evaluated the serum protein levels of bone marrow aplasia markers, EPO and Flt3L, by ELISA. Here, we first compared the levels of EPO and Flt3L in the NRV, NRD (non-irradiated mice treated with CDX-301), RV, and RD groups with the naive group at different time intervals. Subsequently, we also compared those in the RD group with those in the RV group at different time intervals. We found no significant alteration in either EPO or Flt3L in NRV and NRD groups compared with the naive group.

**Figure 7:**
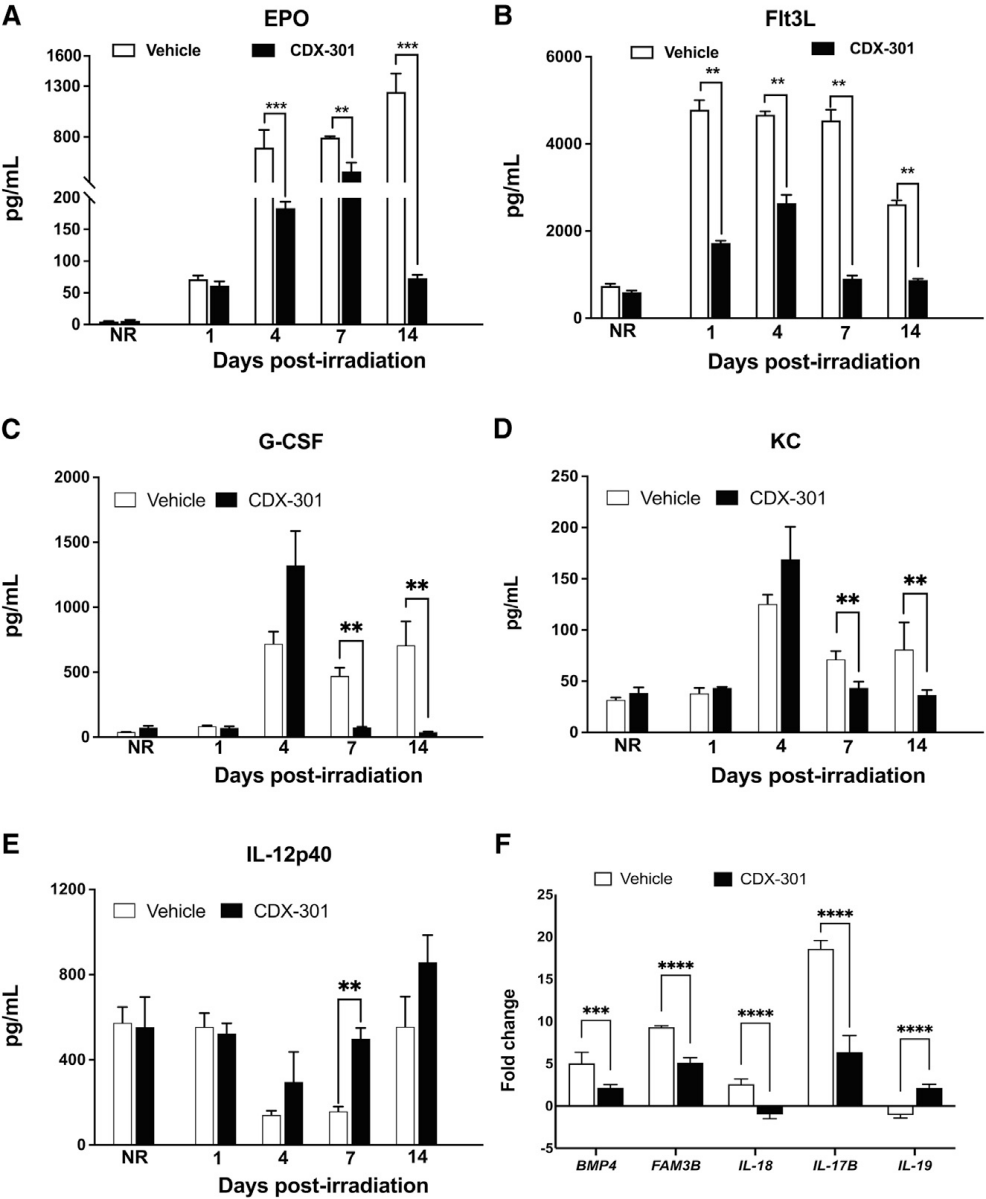
Effect of radiation and impact of pre-treatment with CDX-301 on inflammatory and immune responses (A–E) Bar charts show the longitudinal expression of protein (A) EPO, (B) FLT-3L, (C) G-CSF, (D) KC, and (E) IL-12p40 in serum collected from NRV and NRD, and RV and RD groups on days 1, 4, 7, and 14 post irradiation. (F) Bar chart shows the expression of genes (*BMP4*, *FAM3B*, *IL-17B*, *IL-18*, and *IL-19*) in spleen tissues collected from the RV and RD groups at day 14 post irradiation. Data presented are mean ± standard error of the mean. Significant difference by t test is indicated with asterisks. For EPO and FLT-3L, ∗∗∗p ≤ 0.001, ∗∗p ≤ 0.01; for G-CSF, KC, and IL-12p40, ∗∗p = 0.01–0.04; and for *BMP4*, *FAM3B*, *IL-17B*, *IL-18*, and *IL-19*, ∗∗∗p = 0.0004, ∗∗∗∗p < 0.0001.

Intriguingly, we detected a gradual increase in EPO from days 1 (71.4 ± 14.2 pg/mL), 4 (695 ± 431 pg/mL), and 7 (796 ± 26 pg/mL) to 14 (1,244 ± 449 pg/mL) post irradiation in the RV group compared with the naive group (Figure 7A). Remarkably, we found that pre-treatment with CDX-301 significantly (p ≤ 0.001) reduced EPO from day 4 post irradiation (183 ± 26 pg/mL) and had a maximum decrease at day 14 (72.9 ± 12.9 pg/mL) compared with the RV group (Figure 7A). These data reveal that pre-treatment with CDX-301 alleviated radiation-induced serum EPO levels and provided the ability to protect against radiation-induced stress. In concurrence, an increase in Flt3L on days 1 (4,782 ± 550 pg/mL), 4 (4,672 ± 191 pg/mL), 7 (4,539 ± 608 pg/mL), and 14 (2,617 ± 214 pg/mL) post irradiation were detected in the RV group compared with the naive group. Notably, we found that pre-treatment with CDX-301 significantly (p ≤ 0.01) decreased Flt3L on days 7 (907 ± 148 pg/mL) and 14 (878 ± 74.7 pg/mL) post irradiation in comparison with the RV group (Figure 7B). Thus, pre-treatment with CDX-301 mitigated radiation-induced serum EPO and Flt3L levels and is a potential protective agent against radiation-induced hematopoietic damage.

### CDX-301 mitigates the radiation-induced increased levels of G-CSF and KC, and decreased level of IL-12p40 in murine serum

To evaluate the effect of CDX-301 on the radiation-induced alteration of inflammatory cytokines and growth factors, we assessed the serum level of cytokines and growth factors by Luminex assays. Here, we first compared the protein levels in the NRV, NRD, RV, and RD groups with the naive group at different time intervals. Subsequently, we also compared these in the RD group with those in the RV group. We found no significant alteration in granulocyte colony-stimulating factor (G-CSF), keratinocyte-derived chemokine (KC), and interleukin-12p40 (IL-12p40) in NRV and NRD groups compared with the naive group.

Intriguingly, we detected an increase in G-CSF on days 4 (718 ± 94 pg/mL), 7 (471 ± 62 pg/mL), and 14 (707 ± 183 pg/mL) post irradiation in the RV group compared with the naive group (Figure 7C). Remarkably, we found that pre-treatment with CDX-301 significantly reduced G-CSF on days 7 (74 ± 7 pg/mL, p = 0.012) and 14 (37 ± 7 pg/mL, p = 0.034) post irradiation in comparison with the RV group (Figure 7C). Similarly, KC was also increased on days 4 (125 ± 9 pg/mL), 7 (71 ± 8 pg/mL), and 14 (81 ± 27 pg/mL) post irradiation in the RV group compared with the naive group (Figure 7D). Interestingly, pre-treatment with CDX-301 significantly reduced KC on days 7 (44 ± 6 pg/mL, p = 0.028) and 14 (37 ± 5 pg/mL, p = 0.039) post irradiation in comparison with the RV group (Figure 7D). In concurrence, IL-12p40 levels were reduced on days 4 (141 ± 20 pg/mL) and 7 (158 ± 22 pg/mL) post irradiation in the RV group compared with the NRV and NRD groups (Figure 7E). Notably, pre-treatment with CDX-301 significantly increased IL-12p40 at day 7 (556 ± 142 pg/mL, p = 0.011) post irradiation in comparison with the RV group. CDX-301 mitigated radiation-induced increased levels of G-CSF and KC and decreased levels of IL-12p40, and thus have the potential to provide protection against radiation-induced inflammatory responses.

### CDX-301 modulates the genes associated with MAP kinase and TGF-β pathway

To further assess the effect of pre-treatment with CDX-301 on the radiation-induced alteration of molecular and cellular functions, we examined differential expression of the genes associated with MAP kinase and TGF-β pathways in the spleen samples of RV and RD groups at day 14 post irradiation compared with the NRV group. This was performed by real-time quantitative PCR (qPCR) using a Qiagen RT2 Profiler PCR Array. Subsequently, we also evaluated the differential expression of these genes in the RD group compared with the RV group.

The data revealed upregulation of bone morphogenetic protein 4 (*BMP4*, 5-fold), family with sequence similarity 3 member B (*FAM3B*, 9-fold), *IL-17B* (18.6-fold), and *IL-18* (2.6-fold), and downregulation of *IL-19* (0.9-fold) in the RV group compared with the naive control group (Figure 7F). Notably, we found that CDX-301 significantly decreased expression of *BMP4* (∼2-fold, p = 0.0004), *FAM3B* (∼1.8-fold, p < 0.0001), *IL-17B* (∼2.9-fold, p < 0.0001), and *IL-18* (∼3.5-fold, p < 0.0001), and increased expression of *IL-19* (∼3-fold, p < 0.0001) in comparison with the RV group (Figure 7F). Thus, CDX-301 alleviates radiation-induced increased expression of genes *BMP4*, *FAM3B*, *IL-17B*, and *IL-18* and decreased expression of *IL-19*. These outcomes suggest the role of CDX-301in the regulation of MAP kinase and TGF-β pathways, which are altered because of radiation-induced injury.

## Discussion

Hematopoietic ARS (H-ARS) is an early and major side effect of radiation, which may arise from long-lasting lymphoid cell deficiencies. Identification and development of prophylactic radiation countermeasures and mitigators have the potential for early intervention and could, importantly, prevent clinical consequences of exposure to ionizing radiation. CDX-301 is recombinantly developed (Celldex Therapeutics) from a human protein Flt3L (a hematopoietic cytokine). It is a soluble and non-covalent homodimer that comprises 153 N-terminal amino acids of the extracellular domain of Flt3L. Earlier work demonstrated that Flt3 expression within the CD34^+^ compartment of human hematopoietic stem cells (Flt3^+^CD34^+^ HSC) reconstitute myelopoiesis and lymphopoiesis.^22^ Flt3L treatment boosts immune reconstitution following bone marrow transplantation and corroborates as a potent immune-restorative agent.^38^ Several studies have also demonstrated the role of Flt3L/CDX-301 expression in the reconstitution of myelopoiesis and lymphopoiesis and the immune system during HSC transplantation (HSCT).^20^^,^^24^^,^^25^^,^^26^ Importantly, CDX-301 has been shown to be safe in toxicology studies and has been used in a phase 1 clinical trial for HSCT and cancer immunotherapy.^20^^,^^21^ These studies clearly demonstrate the potential of Flt3L/CDX-301 as a drug for conquering long-lasting lymphoid cell deficiencies.

Here, with analyses of global miRNA expression profiles and their associated pathways in the serum and the spleen tissue samples from mice with or without administration of CDX-301 24 h prior to TBI, we demonstrate that the prophylactic efficacy of CDX-301 mediates through the attenuation of radiation-induced aberrant expression of miRNAs and several molecular and cellular mechanisms. Previously we demonstrated the radioprotective and radiomitigative effectiveness of CDX-301, whereby the protection and recovery of both myeloid and lymphoid cells in circulation and within tissue is observed against radiation-induced injuries and damage.^30^ In that study, we identified that a single subcutaneous dose of CDX-301 (400 μg/kg) administered 24 h prior to Co-60 gamma TBI afforded an 88% increase in 30-day survival in CD2F1 male mice. We also demonstrated that CDX-301 accelerates recovery from irradiation-induced peripheral blood cytopenia and expedites recovery of bone marrow stem and progenitor cells as well as increasing splenic CD3^+^ T cells, specifically CD4^+^ Th cells. Our data establish CDX-301 as a promising prophylactic agent against exposure to radiation. In line with this, our current study demonstrates the radioprotective molecular and cellular mechanisms of CDX-301 through which it increases survival, averts injury, and mitigates H-ARS.

miRNAs are short non-coding RNAs involved in the regulation of nearly all cellular processes including DNA repair, apoptosis, necrosis, cell-cycle arrest, and survival. Under normal conditions, the expression of miRNAs is precisely coordinated through several mechanisms such as genetic or epigenetic changes, transcription factor recruitment, and the appropriate role of effectors involved in the miRNA biogenesis pathway.^39^^,^^40^ Deviation in any of these mechanisms could lead to changes in miRNA expression. Exposure to radiation dysregulates several cellular processes including DNA repair, apoptosis, necrosis, cell-cycle arrest, and survival.^41^ To counteract these cellular responses, the cell initiates a number of cellular changes, including change in miRNA expression in a dose-, time-, and tissue-specific manner.^42^^,^^43^^,^^44^^,^^45^^,^^46^^,^^47^^,^^48^ Our current analyses show that exposure to radiation alters miRNA expression in both murine serum and spleen in a time- and tissue-dependent manner. In serum samples from the RV group, miRNA profiling reveals a total of 50, 35, 46, and 84 miRNAs with altered expressions on days 1, 4, 7, and 14 post irradiation, respectively, in response to radiation exposure compared with the NRV group. Similarly, in mouse spleen tissues expression of a total of 127, 150, 121, and 94 miRNAs were altered on days 1, 4, 7, and 14 post irradiation, respectively, in response to radiation exposure (RV compared with NRV group). Furthermore, we also find that there is a variation in expression pattern and levels among those miRNAs that are altered in all four groups Rd1V, Rd4V, Rd7V, and Rd14V. Earlier, Ilnytskyy et al. reported that whole-body radiation exposure leads to significant changes in the expression miRNAs in murine spleen and thymus tissue.^42^ The alteration in serum miRNA expression has been revealed in mice exposed to TBI.^43^ Time-dependent changes in miRNA expression have been demonstrated in serum, plasma, and whole blood of mice exposed to TBI.^44^^,^^45^^,^^46^^,^^47^ Tissue-specific changes of miRNAs in murine liver, heart, and testis have been demonstrated in mice exposed to TBI.^48^

In the analysis of the efficacy of drugs, the role of miRNAs has achieved substantial consideration and opened a new field known as miRNA pharmacogenomics,^34^^,^^35^^,^^36^^,^^37^ which is the study of drug administration effect on the expression of miRNAs and their associated target genes and pathways. Small molecules or drugs could regulate miRNA biogenesis and expression at all three stages (before, during, and after) of transcription.^49^^,^^50^^,^^51^ Here, we decipher the relationship between the promising prophylactic radiation countermeasure CDX-301 and its treatment response mediated by altered expression of miRNAs in serum and spleen samples from mice exposed to TBI. This is the first study to report that CDX-301 regulates the expression of miRNAs in the serum and spleen from irradiated mice in a time- and tissue-dependent manner. Notably, our analyses show that pre-treatment with CDX-301 prevents the radiation-induced dysregulation of miRNA biogenesis and restores the global miRNA levels in both the serum and spleen from irradiated mice. A previous study suggests that global suppression of miRNA levels (inhibition of miRNA biogenesis), attained in endothelial cells through the silencing of miRNA processing enzymes AGO2 or DICER, amplifies the level of cell death after irradiation.^52^ Decreased expression of DROSHA and DICER has been found in radiosensitive cell lines compared with that in resistant cell lines.^53^ In a recent study, it has been suggested that radiation directly regulates miRNA biogenesis or some of its processing enzymes.^46^ Subsequently, in our upstream regulator analysis of differentially expressed miRNAs in the RV and RD groups, we find that in response to radiation (RV group), enzymes of miRNA processing such as AGO2, DGCR8, and DICER1 are inhibited in serum and/or spleen samples on days 7 and/or 14 post irradiation. Interestingly, our analysis demonstrates activation of the AGO2, DGCR8, and DICER1 on days 7 and/or 14 post irradiation in serum and/or spleen samples from the RD group. Together, these data suggest that pre-treatment with CDX-301 prevents the radiation-induced dysregulation of miRNA biogenesis.

Strikingly, our comparative analyses of RV and RD groups also reveal that pre-treatment with CDX-301 prevents radiation-induced aberrant alteration of miRNA expression in irradiated mouse serum as well as spleen in a time- and tissue-dependent manner. Both in serum and spleen tissues we find that most of the miRNAs whose expression significantly decreased in response to radiation have increased expression by pre-treatment with CDX-301 on their respective days. This suggests that CDX-301 suppresses radiation-induced decrease in miRNA expression in the serum and spleen from irradiated mice. Concurrently, we find that CDX-301 suppresses radiation-induced upregulation of miRNAs in the serum and spleen of irradiated mice in a time- and tissue-dependent manner. Overall, these results indicate that pre-treatment with CDX-301 prevents radiation-induced alteration of miRNA expression (either downregulation or upregulation) in the serum as well as the spleen tissues of irradiated mice. Our results corroborate studies that report radiation-induced restoration of serum miRNAs in mice pre-treated with amifostine before TBI. The same study demonstrated that downregulated miRs miR-187-3p, miR-194-5p, and miR-27a-3p were upregulated; similarly, radiation-induced upregulated serum miRNAs such as miR-30a-3p and miR-30c-5p were downregulated in mice treated with amifostine before TBI and correlated with survival of irradiated mice.^43^ In another study, radiation-induced upregulated tissues and serum miRNAs such as miR-30b and miR-30c were downregulated in mice treated with delta-tocotrienol (DT3) before TBI and correlated with the survival of irradiated mice.^54^ Furthermore, it has been shown that radiation-induced downregulated spleen miRNAs such as miR-15b and miR-92b, and radiation-induced upregulated spleen miRNAs such as miR-27b, miR-34a, miR-99b, miR-125b, miR-126, miR-130a, miR-143, and miR-145b, were suppressed by pre-treatment with GT3 in irradiated mice, resulting in a reduction of splenic damage, protection of the hematopoietic system, and increased survival.^15^ Recent studies have demonstrated the upregulation of serum and spleen miRNAs such as miR-296-5p and miR-328-3p in mice treated with romiplostim in a radiation-induced leukemogenesis model, while miR486-5p is downregulated.^55^ The authors suggest the involvement of romiplostim in the biogenesis of miRNAs that mitigate radiation-induced leukemia.

Remarkably, we found miRNAs that were significantly altered in expression (miR-125a-3p, miR-125b-5p, miR-135a-5p, miR-142-3p, miR-15, miR-155, miR-17, miR-34, miR-486-5p, and miR-680) in both the serum and spleen samples. The dysregulation of the same miRNAs in both serum and spleen suggests a potential global effect of radiation on common signaling pathways. Intriguingly, IPA analyses identify the HOTAIR regulatory pathway as one of the most affected canonical pathways in the serum and spleen tissues of the RV and RD groups. Further analyses of differentially expressed miRNAs (let-7, miR-130, miR-148, miR-29, miR-331-3p, miR-7, and miR-8) involved in HOTAIR regulatory pathways indicate that pre-treatment with CDX-301 can significantly restore the expression levels of most of these miRNAs. This suggests that pre-treatment with CDX-301 prevents radiation-induced dysregulated expression (downregulation or upregulation) of miRNAs involved in the HOTAIR regulatory pathway. In response to radiation, the crucial role of long non-coding RNA HOTAIR has been shown in a variety of tissue models. For example, overexpression of HOTAIR increases cellular radioresistivity through the modulation of WIF-1, HIF-1α, and p21 in pancreatic ductal adenocarcinoma cells, cervical cancer cell lines, and HeLa and C33A cells, respectively.^56^^,^^57^^,^^58^ In breast cancer cell lines, knockdown of HOTAIR increases cellular radiosensitivity and promotes DNA damage and cell-cycle arrest by suppression of miR-218.^59^ In another study of breast cancer cell lines, the role of HOTAIR in the regulation of cellular radiosensitivity has been reported through the sponging of miR-449b-5p.^60^ Furthermore, in a Göttingen minipig model, radiation-responsive mRNA expression profiles predicted repression of the HOTAIR regulatory pathway at day 7 post irradiation.^61^ Consistently, our upstream regulator analysis shows that in response to radiation, HOTAIR is activated on days 7 and/or 14 post irradiation in serum and/or spleen samples of the RV group. Interestingly, in the samples from the RD group (pre-treated with CDX-301), we find inhibition of HOTAIR on days 7 and/or 14 post-irradiation in serum and/or spleen samples of the RD group. These data along with earlier reports suggest that pre-treatment with CDX-301 prevents radiation-induced activation of HOTAIR and may diminish cellular radiosensitivity and suppress DNA damage and cell-cycle arrest.

Radiation induces dysregulation in several cellular functions including cell proliferation, death, invasion and metastasis, genomic stability, energy metabolism, inflammation, and immunogenicity.^62^^,^^63^ Here, we also performed disease and cellular function analysis for differentially expressed miRNAs in the serum and spleen samples of the RV and RD groups. The analysis shows that in response to radiation, several cellular functions such as the cell proliferation of lymphoma cell lines, inflammatory response, and development of epithelial tissue are activated, and the cytokines of ventricular monocytes, the proliferation of pancreatic cell lines, and epithelial-mesenchymal transition of tumor cell lines are inhibited on days 7 and 14 post irradiation in serum samples and on day 14 post irradiation in spleen samples of RV. Interestingly, we find that pre-treatment with CDX-301 prevents radiation-induced dysregulation of cellular functions. Consistently, our analysis demonstrates inhibition of the cell proliferation of lymphoma cell lines, inflammatory response and development of epithelial tissue, activation of the cytokines of ventricular monocytes, proliferation of pancreatic cell lines, and epithelial-mesenchymal transition of tumor cell lines on days 7 and 14 post irradiation in serum samples and on day 14 post irradiation in spleen samples of the RD group. In addition, the inflammation of absolute anatomical region and inflammation of organ pathways, which are analyzed to be activated in the RV group, are inhibited in serum of mice pre-treated with CDX-301. Furthermore, the differentiation of Th17 cells, which is inhibited in serum samples of the RV group, is activated in the RD group. Overall, these findings demonstrate that pre-treatment with CDX-301 modulates various disease and cellular functions in both serum and spleen.

Analyses of the differentially regulated miRNAs and associated pathways reveal that the prophylactic efficacy of CDX-301 is mediated through the attenuation of radiation-induced aberrant expression of miRNAs, inflammatory responses, and molecular and cellular mechanisms. To assess the effect of pre-treatment with CDX-301 on the radiation-induced damage, alteration of inflammatory cytokines and growth factors, and molecular and cellular functions, we evaluated the protein level of EPO, Flt3L, and inflammatory cytokines and growth factors, and expression of genes associated with the MAP kinase and TGF-β pathways. The increased production of hematopoietic cytokines such as EPO and Flt3L has been shown to be induced by exposure to radiation.^8^^,^^15^^,^^64^^,^^65^ We find a gradual increase in EPO levels on days 1, 4, 7, and 14 post irradiation in the RV group. Furthermore, pre-treatment with CDX-301 before exposure to radiation significantly reduces EPO levels from day 4 post irradiation onward, with the largest reduction observed on day 14 post irradiation, compared with the RV group. This suggests a protective role of CDX-301 against radiation-induced stress. In concurrence, we find a radiation-induced significant increase in levels of Flt3L, a radiation-specific biomarker for bone marrow aplasia, which is attenuated by pre-treatment with CDX-301. Consistent with our findings, the reduction of radiation-induced serum EPO and Flt3L levels has been demonstrated in mice administered either GT3 or BBT-059 prior to radiation exposure.^8^^,^^15^ In addition, the analysis of upstream regulators also indicates that in response to radiation, Flt3 is activated in both the serum and spleen samples from the RV group and is inhibited in RD group. These data reveal that pre-treatment with CDX-301 mitigated radiation-induced serum EPO and Flt3L levels and has a pronounced protective effect on radiation-induced hematopoietic damage.

Cytokines/chemokines and growth factors play a major role in radiation-induced immune responses.^66^ We find an increase in G-CSF and KC on days 4, 7, and 14 post irradiation in the RV group, which is significantly reduced by pre-treatment with CDX-301. In concurrence, IL-12p40 has decreased levels on days 4 and 7 post irradiation in the RV group, which is significantly increased by pre-treatment with CDX-301. It has been shown earlier that various cell types including endothelial cells and T lymphocytes synthesize glycoprotein hematopoietic growth factors. G-CSF is one of these factors essential for the differentiation of progenitor cells into neutrophils in bone marrow.^66^ We have previously shown that pre-treatment of mice with CDX-301 prior to TBI accelerated the production of neutrophils and resulted in increased survival.^30^ The protective role of G-CSF and other cytokines on the survival of bone marrow progenitor cells after exposure to ionizing radiation has also been reported.^66^ Consistently, our data reveal that pre-treatment with CDX-301 mitigated radiation-induced increase in the levels of G-CSF and KC and decrease in the level of IL-12p40, thus providing protection against radiation-induced inflammatory response and hematopoietic damage.

The MAP kinase pathway transduces signals from the cell membrane to the nucleus in response to various stimuli and participates in several intracellular signaling pathways.^67^ TGF-β plays an essential role in extracellular matrix remodeling, cell mobility, and modulation of the immune response. Peripheral blood TGF-β levels can be used to assess the risk of radiation-induced lung damage.^68^ We find that pre-treatment with CDX-301 of mice exposed to TBI counteracts the upregulation of *BMP4*, *FAM3B*, *IL-17B*, and *IL-18*, and downregulation of *IL-19* genes, associated with MAP kinase and TGF-β pathways. Previously it has been shown that in a mouse inflammation model EPO stimulates spleen BMP4-dependent stress-induced erythropoiesis and reduces anemia.^69^ Three members of the interleukin family (IL-17B, IL-18, and IL-19) are also found to be significantly modulated by drug treatment. Interferon-γ treatment of islets has been shown to induce upregulation of FAM3B gene expression, which is the likely cause of cytokine-induced β-cell dysfunction and death.^70^ IL-18 plays a key role in radiation-induced cell and tissue damage and dysfunction. Circulating IL-18 has been shown to be a biomarker of exposure to TBI in three animal models, namely mice, minipigs, and non-human primates.^71^^,^^72^ Administration of human rIL-17B has been shown to promote neutrophil migration in healthy mice.^73^ Moreover, IL-17 family members induce inflammatory cytokines not only through activated T cells but also through activated monocytes/macrophages, which are the primary cells in the bone marrow that are damaged by exposure to radiation.^73^ Small et al. have reported that the gene encoding the cytokine IL-19, which is associated with inflammatory disorders and cancer, was induced in mouse and human cells in response to various cellular insults, including DNA damage.^74^ These findings reveal that pre-treatment with CDX-301 alleviates radiation-induced increased expression of genes *BMP4*, *FAM3B*, *IL-17B*, and *IL-18* and decreased expression of *IL-19*. These outcomes suggest that pre-treatment with CDX-301 regulates MAP kinase and TGF-β pathways, and offers protection against radiation-induced hematopoietic damage and alteration of molecular and cellular functions.

In conclusion, our results indicate that CDX-301 administration 24 h prior to TBI averts the radiation-induced lethal response by preventing dysregulation of miRNA biogenesis, thus attenuating radiation-induced dysregulation of cellular functions. These results along with earlier studies demonstrate that CDX-301 is an efficient radioprotective drug and has a pronounced effect on preventing radiation-induced DNA damage and cell-cycle arrest, diminution of cellular radiosensitivity, and protection from hematopoietic damage. Further pre-clinical studies are required to establish the radioprophylactic mechanism of CDX-301 and to develop an optimal therapeutic protocol for use in humans to prevent H-ARS.

## Materials and methods

### Mice

Ten- to eleven-week-old male CD2F1 mice (Envigo, Indianapolis, IN, USA) were housed (four per cage) in an air-conditioned facility at the Armed Forces Radiobiology Research Institute (AFRRI) (Bethesda, MD), accredited by the Association for Assessment and Accreditation of Laboratory Animal Care International. The holding rooms for mice were maintained at 21°C ± 2°C with 10–15 hourly cycles of fresh air and relative humidity of 50% ± 10%. Mice were kept in quarantine for 2 weeks and were used after microbiology, serology, and histopathology examination of representative samples ensured the absence of *Pseudomonas aeruginosa* and common murine diseases.^30^ All animals were provided with certified rodent food and acidified water with hydrochloric acid (pH 2.5–3.0) *ad libitum*. All animal procedures were reviewed and approved by the AFRRI Institutional Animal Care and Use Committee using the principles outlined in the National Research Council’s Guide for the Care and Use of Laboratory Animals.^9^^,^^13^^,^^30^

### CDX-301 formulation and administration

CDX-301, manufactured under current good manufacturing practices, received from Celldex Therapeutics, was formulated in normal sterile saline (0.9% NaCl) before use as described earlier.^30^ Saline was purchased from the Uniformed Services University of the Health Sciences pharmacy and used as vehicle control. Based on the findings of the optimum dose and time of CDX-301 administration prior to TBI essentially as described previously,^30^ two groups of CD2F1 male mice (n = 12 per group) were subcutaneously, at the nape of the neck, administered either with 0.1 mL of CDX-301 (400 μg/kg) or its vehicle saline 24 h prior to TBI (radiation dose 7 Gy at the rate of ∼0.6 Gy/min). Two additional control groups (non-irradiated CDX-301 and non-irradiated vehicle) of CD2F1 male mice (n = 3 per group) were also included in the study.

### Irradiation

Mice were irradiated bilaterally (simultaneously) at a non-lethal dose of 7 Gy at an estimated dose rate of 0.6 Gy/min in the AFRRI’s Cobalt-60 γ-irradiation facility. An alanine/electron spin resonance (ESR) dosimetry system (American Society for Testing and Material Standard E 1607) has been used to quantify the dose rates in the cores of acrylic phantoms as described earlier.^30^ ESR signal was determined by calibration curve based on standard calibration dosimeters provided by the National Institute of Standards and Technology (Gaithersburg, MD) as reported earlier.^11^ Following standard dosimetry at AFRRI, the calibration curve was confirmed by inter-comparison with the National Physical Laboratory in the United Kingdom.^11^ The only corrections applied to the measured dose rates in phantoms were for the decay of the γ-irradiation source and for a small difference in mass-energy absorption coefficients for water and soft tissue. The radiation field was uniform within ±2%.^30^ After irradiation, animals were returned to their cages and monitored two times a day (morning and evening). Since mice were irradiated at a non-lethal dose of 7 Gy, no animals were moribund during the duration of the study.

### Blood and spleen harvest

The blood and spleen samples from either mouse pre-treated with CDX-301 or its vehicle control were collected at four time points: days 1, 4, 7, and 14 post TBI (n = 3 per time point and group). The blood samples were collected on specified days post irradiation under anesthesia by inferior vena cava, placed in a serum separator tube for clotting, and finally centrifuged; supernatants were then stored at −80°C. The spleen samples were harvested, snap-frozen, and stored at −80°C for future mechanistic studies.

### Total RNA extraction

Approximately 50 mg of the frozen spleen was homogenized by brief sonication in ice, and total RNA was extracted using mirVana total RNA isolation kits (Life Technologies, Frederick, MD) following the manufacturer’s protocol. Similarly, total RNA from serum was isolated using a mirVana PARIS RNA purification kit (Life Technologies). The quality and concentration of spleen and serum RNAs were assessed using the NanoDrop spectrophotometer (NanoDrop Technologies, Wilmington, DE). All RNA samples had 260/280 nm absorbance ratios of 1.8–2.0.

### Murine microRNA analysis

miRNA analysis was performed following standard methods published by the vendor. Multiplex reverse transcription (RT) was performed with a TaqMan MicroRNA Reverse Transcription Kit (Applied Biosystems). Following RT, pre-amplification was performed using Megaplex PreAmp Primers and TaqMan Preamp Master Mix (2×). The PreAmp product was used to prepare PCR reaction mix using TaqMan Universal PCR Master Mix, No AmpErase UNG (2×). One hundred microliters of the RT reaction-specific PCR reaction mix was loaded into the corresponding fill ports of the TaqMan Low-Density Rodent MicroRNA Panel v2.0 and run using default thermal-cycling conditions on the 7900HT Fast Real-time PCR System (Applied Biosystems).

Data analysis was carried out online with Expression Suite Software v1.0.3 (Thermo Fisher Cloud, Applied Biosystems, Thermo Fisher Scientific, Waltham, MA). The relative quantification (Rq) method with global normalization was used to calculate the comparative cycle threshold Ct (2^−ΔΔCt^) and determine relative fold changes of each miRNA in either mice treated with vehicle 24 h prior to TBI (RV) or mice treated with CDX-301 24 h prior to TBI (RD) versus non-irradiated vehicle-treated mice (NRV) at different time intervals. Further analysis was performed to compare the miRNAs that were significantly altered (at various time intervals) in the RD group with those in the RV group. The Venn diagrams were generated using the online tool https://bioinformatics.psb.ugent.be/webtools/Venn/. The heatmaps were constructed using Clustvis, a web tool for visualizing clustering of multivariate data using principal component analysis and heatmap (https://biit.cs.ut.ee/clustvis/).^75^

### Pathway analyses

*In silico* analysis of differentially expressed miRNAs (fold change >0.5 and <1.2) in the serum and spleen of mice from the various groups (control as well as pre-treated with CDX-301) was performed using IPA (Qiagen, https://www.qiagenbioinformatics.com/products/ingenuity-pathway-analysis). The analyses were based on experimentally observed and highly predicted data from the Ingenuity Knowledge Base data sources. The top canonical pathways, significant (p < 0.05) regulators, diseases and functions, and gene networks related to our study were identified, and the differentially expressed genes with specific functions were categorized. These genes were then used to generate functional networks between miRNAs and their target genes as well as regulatory molecules.

### ELISA

Biomarkers of bone marrow hyperplasia, EPO, and Flt3L, were measured in serum. MiceEPO Quantikine ELISA and mouse/Rat Flt3L Quantikine ELISA kits were purchased from R&D Systems (Minneapolis, MN). The cytokine detection limits were >47 pg/mL and >5 pg/mL for EPO and Flt3L ELISAs, respectively. The quantitative levels of EPO and Flt3L were evaluated from serum samples collected on days 1, 4, 7, and 14 post TBI following standard protocols.^8^ Some samples had to be diluted to keep the readings within the assay range specified by the vendor. Data represented are mean ± standard error of the mean for n = 3 mice per group with replicates.

### Luminex cytokine assays

Levels of circulatory cytokines were measured using a Bio-Plex Mouse Cytokine 23-Plex Panel (Bio-Rad, Hercules, CA) assay unit according to the manufacturer’s instructions. The Luminex cytokine assays are multiplex magnetic bead-based assays designed to quantitate multiple cytokines in the Bio-Ple×200 (Bio-Rad), a dual-laser flow analyzer. Mouse serum samples were analyzed for interleukin-1β (IL-1β), IL-6, IL-10, IL-12 (p70), G-CSF, granulocyte-macrophage colony-stimulating factor (GM-CSF), KC, and tumor necrosis factor α.

The Luminex assay protocol was followed as previously described.^66^ In brief, 50 μL of magnetic beads was added to desired wells in a 96-well plate after vertexing for 15–20 s at medium speed and then washed two times in a Bio-Plex Pro wash station with a 100 μL of wash buffer. Next, 50 μL of the diluted standard or serum samples (1:4 with the diluent provided) were added to each well. The plate was then washed in the Bio-Plex Pro wash station, and the diluted biotinylated detection antibody was added to each well. After incubation for 1 h, the plate was washed three times in the Bio-Plex Pro wash station to remove excess detection antibodies and streptavidin-phycoerythrin. After final incubation and washing, the fluorochrome bound to magnetic beads was immediately read on the Bio-Ple×200 system, and Bio-Plex Manager Version 5.0 was used for data acquisition.

### Real-time quantitative PCR

RNA was reverse transcribed into cDNA (RT2 First Strand Kit; Qiagen), and the cDNA was used for qPCR analysis. Gene expression in MAP kinase (catalog #PAMM-061ZA) and TGF-β (catalog #PAMM-035ZA) pathways were analyzed by real-time qPCR using a Qiagen RT2 Profiler PCR Array (https://geneglobe.qiagen.com/us/product-groups/rt2-profiler-pcr-arrays) and RT2 SYBR Green Master Mixes. Analyses were performed on https://geneglobe.qiagen.com/us/analyze. ΔCt values were calculated by subtracting average Ct values of housekeeping genes (β2-microglobulin, *B2M*; glyceraldehyde-3-phosphate dehydrogenase, *GAPDH*; actin, beta, *ACTB*; ribosomal protein, large, P0, *RPLP0*; and hypoxanthine guanine phosphoribosyl transferase 1, *HPRT1*). Data were evaluated by the ΔΔCt method against NRV control (n = 4), and fold changes in gene expression were calculated after normalizing data to the array’s housekeeping gene pool.

### Statistical analysis

Comparisons of each miRNA expression between groups at different time intervals were analyzed using a Student’s t test. Heatmaps were clustered using Euclidean distance for similarity measurements and average linkage as the association method. Pathway analyses of differentially expressed miRNAs in groups at different time intervals were analyzed using Fisher’s exact test. Analysis of variance or t test was used to determine if there was a significant difference between the two groups. Data with a p value of <0.05 were considered statistically significant.

## Data Availability

The datasets generated during and/or analyzed during the current study are available from the corresponding author on reasonable request.

## Supplemental information

Document S1. Figure S1 and Tables S1–S3

Document S2. Article plus supplemental information
