# Supplementary material for: CDX-301 prevents radiation-induced dysregulation of miRNA expression and biogenesis
Source: Mol Ther Nucleic Acids. 2022 Nov 15;30:569–84. doi: 10.1016/j.omtn.2022.11.010 (PMC9703457; doi:10.1016/j.omtn.2022.11.010)
Supplement: Document S1. Figure S1 and Tables S1–S3 [file mmc1.pdf]

OMTN, Volume *30*

Supplemental information

CDX-301 prevents radiation-induced dysregulation of miRNA expression and biogenesis

Dharmendra Kumar Soni, Vidya P. Kumar, Shukla Biswas, Gregory P. Holmes-Hampton, Sharmistha Bhattacharyya, Lawrence J. Thomas, Roopa Biswas, and Sanchita P. Ghosh

Table S1. Upstream regulators and their Z score in the serum and spleen samples from mice treated with vehicle 24 h prior to irradiation (RV) group and mice treated with CDX-301 24 h prior to irradiation (RD) group on days 7 and 14 post-irradiation.

	Serum (Z score)				Spleen (Z score)	
**Symbol**	**Rd7V**	**Rd7D**	**Rd14V**	**Rd14D**	**Rd14V**	**Rd14D**
AGO2	-1.60	3.65	-1.69	3.41	-2.96	2.76
DGCR8	N/A	N/A	N/A	N/A	-2.22	1.31
DICER1	0.46	2.79	-0.61	1.29	-1.35	2.11
E2F1	-1.69	1.34	-0.54	2.05	-2.65	1.93
E2F2	-1.97	N/A	-0.88	N/A	-2.20	1.65
E2F3	-2.20	2.19	-1.29	2.19	-2.77	2.54
EPHB6	-1.17	1.48	-0.11	1.11	-1.58	1.90
Gnasas1	1.35	-1.94	1.83	-1.74	2.38	-2.04
HOTAIR	N/A	N/A	N/A	N/A	N/A	-2.00
REST	N/A	N/A	N/A	N/A	N/A	-2.22
SSB	-2.33	3.25	-1.85	3.25	-3.03	1.97

Rd7V and Rd14V = mice treated with vehicle; Rd7D and Rd14D = mice treated with CDX-301; 24 h prior to radiation exposure and samples collected at post-irradiation days 7 and 14.

Table S2. Expression of miRNAs associated with regulation of upstream regulators in the serum and spleen samples from mice treated with vehicle 24 h prior to irradiation (RV) group and mice treated with CDX-301 24 h prior to irradiation (RD) group on days 7 and 14 post-irradiation.

	**Serum (Expr Fold Change)**				**Spleen (Expr Fold Change)**	
**Symbol**	**Rd7V**	**Rd7D**	**Rd14V**	**Rd14D**	**Rd14V**	**Rd14D**
let-7	-15.15	7.39	-8.33	4.25	-4.67	-2.44
miR-10						6.01
miR-130	-3.46	49.21	-4.35	7.04		4.96
miR-143	-5.92		-9.62		-1.94	2.27
miR-146		6.09	-9.43		2.45	
miR-148		4.80				6.21
miR-15	-40.00		12.27		-7.09	2.26
miR-150			-14.08		-8.13	-23.26
miR-154	1856.74		11961.41		36.62	7.29
miR-155	9.23	6.54	5.93	3.18	-4.78	-6.02
miR-17	-7.75	199.23	-3.02	10.93	-9.35	13.69
miR-181						2.51
miR-19		2.45		2.30	-3.41	8.86
miR-191	-3.51		-18.87	2.82		-2.04
miR-25					-2.31	2.22
miR-29		34.83			-6.17	2.30
miR-331-3p	-6.41		-32.26		-1.76	-2.82
miR-34			6.14			11.44
miR-7	-1.92		-34.48	7.14		
miR-8	6.03	4.60	-6.85	-3.06	-2.89	-3.22

Rd7V and Rd14V = mice treated with vehicle; Rd7D and Rd14D = mice treated with CDX-301; 24 h prior to radiation exposure and samples collected at post-irradiation days 7 and 14.

Table S3. Expression of miRNAs associated with regulation of diseases and cellular functions in the serum and spleen samples from mice treated with vehicle 24 h prior to irradiation (RV) group and mice treated with CDX-301 24 h prior to irradiation (RD) group on days 7 and 14 post-irradiation.

	**Serum (Expr Fold Change)**				**Spleen (Expr Fold Change)**	
**Symbol**	**Rd7V**	**Rd7D**	**Rd14V**	**Rd14D**	**Rd14V**	**Rd14D**
let-7	-15.15	7.39	-8.33	4.25	-4.67	-2.44
miR-10						6.01
miR-122		1197.54		15.17		
miR-130	-3.46	49.21	-4.35	7.04		4.96
miR-132						2.72
miR-140	-17.86			5.38	-5.81	2.57
miR-143	-5.92		-9.62		-1.94	2.27
miR-146		6.09	-9.43		2.45	
miR-148		4.80				6.21
miR-15	-40.00		12.27		-7.09	2.26
miR-150			-14.08		-8.13	-23.26
miR-154	1856.74		11961.41		36.62	7.29
miR-155	9.23	6.54	5.93	3.18	-4.78	-6.02
miR-17	-7.75	199.23	-3.02	10.93	-9.35	13.69
miR-181						2.51
miR-185		12.66		9.77		3.37
miR-187			-23.26		-2.87	-20.41
miR-19		2.45		2.30	-3.41	8.86
miR-193		2.00	-2.72	4.54	3.12	-1.94
miR-203	5.51	8.60				
miR-204					-3.10	1.70
miR-221	-4.76	2.64	-7.19	2.46	-1.53	3.55
miR-223		4.49	-4.59	5.32	-6.90	6.16
miR-23						3.31
miR-25					-2.31	2.22
miR-26					-3.02	1.79
miR-27						5.06
miR-29		34.83			-6.17	2.30
miR-30		18.35			-17.24	
miR-31		19.50		12.61		
miR-320						-4.46
miR-322						3.02
miR-34			6.14			11.44
miR-383					-2.88	-3.48

miR-431					7.02	1.53
miR-449	34.12	-3.40	139.36	10.30	-2.30	-2.52
miR-455						1.82
miR-484	-11.90		-25.00		-2.20	-3.08
miR-500						-2.39
miR-503					1.69	2.61
miR-598					-19.61	3.10
miR-8	6.03	4.60	-6.85	-3.06	-2.89	-3.22

Rd7V and Rd14V = mice treated with vehicle; Rd7D and Rd14D = mice treated with CDX-301; 24 h prior to radiation exposure and samples collected at post-irradiation days 7 and 14.

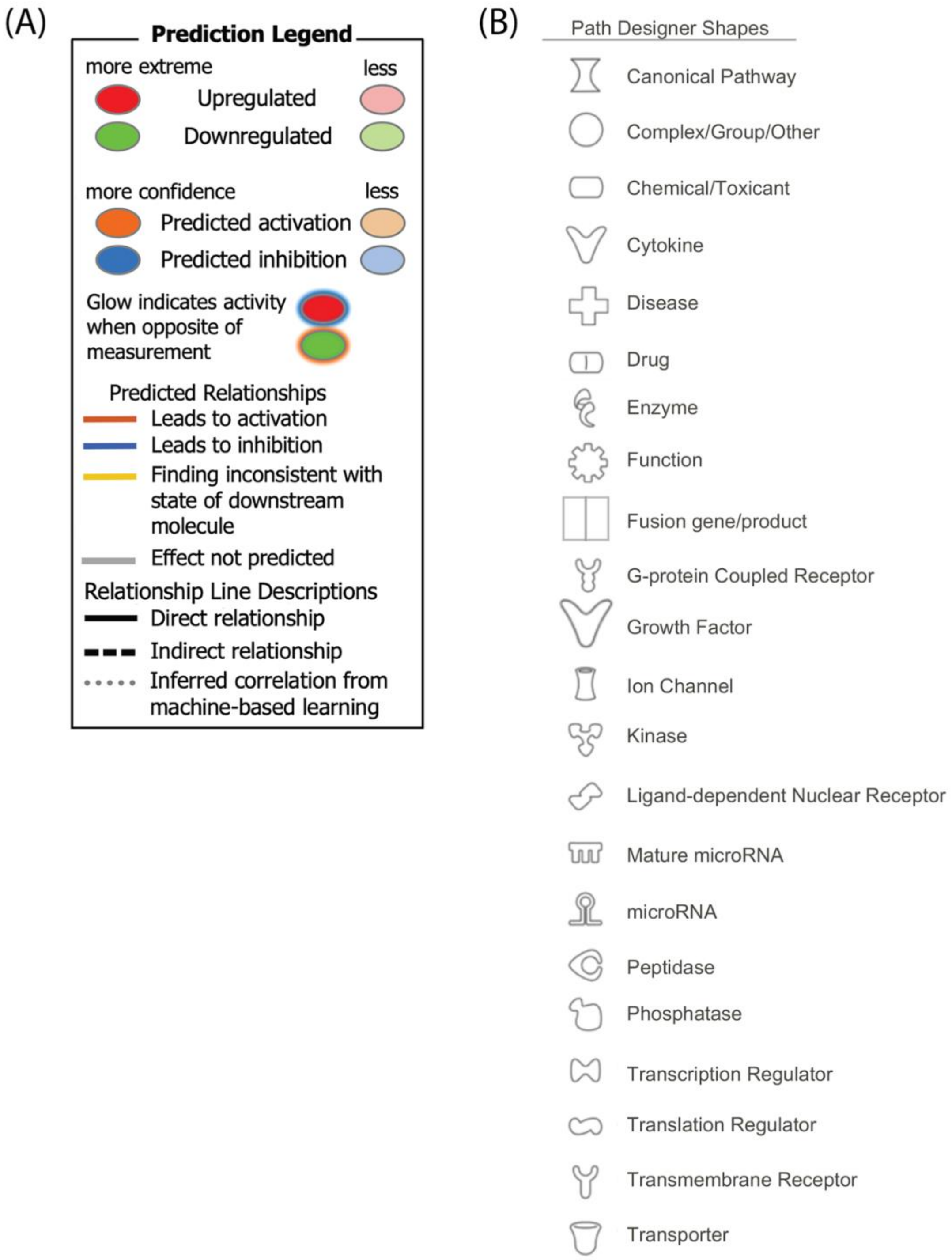

Figure S1. (A) The color scheme indicates the value of the differential expressions. **(B)** The shapes indicate the type and function.
